# Clinical applications of internal fixation via the volar approach with pronator quadratus preservation for distal radius fractures

**DOI:** 10.55730/1300-0144.5421

**Published:** 2022-04-02

**Authors:** Jie ZHONG, Xiaohui LI, Fuxin LV, Naiyue CAO, Dangke LI

**Affiliations:** Department of Traumatic Orthopaedics, Qilu Hospital (Qingdao) of Shandong University, Qingdao, China

**Keywords:** Distal radius fractures, pronator quadratus, clinical applications, preservation, volar approach

## Abstract

**Background/aim:**

The purpose of this study is to investigate the success rate of volar plate treatment of distal radius fractures with preservation of the pronator anterior muscle; the incidence of complications, such as infection, vascular nerve injury, and tendon injury; fracture healing rate; and changes in muscle anatomy.

**Materials and methods:**

The Henry approach was adopted to treat distal radius fractures with pronator quadratus (PQ) preservation on a trial basis. Between June 2015 and January 2017, 46 cases of distal radius fractures were considered according to the Arbeitsgemeinschaft für Osteosynthesefragen (AO) classification. The PQ was preserved, the distal margin was exposed, and a fracture reset was completed by pulling the muscles toward the near side. The K-wires were temporarily fixed, and the plate was placed by a plate channel. The mean operation duration was 52 min and the average blood loss was approximately 30 mL. There were no implant failures, adhesions requiring tenolysis, and tendon rupture. No patient developed carpal tunnel syndrome. All fractures healed without infection, radial artery injury, nerve damage, tendon rupture, and nonunion. A guider was applied to implant a screw under the muscle.

**Results:**

In total, 46 patients with PQ preservation between ages 29 to 52 were performed distal radius fracture surgery. AO classification revealed that there were four cases of type A, seven cases of type B3, 10 cases of type C1, 13 cases of type C2, and 12 cases of type C3. For most fractures, such as Types A, B3, C1, C2, and C3, the fracture sites were located around the muscle distal margin. Thus, slight pulling of the muscles to the near side can reveal the fracture, and surgery with PQ preservation can be implemented. The postoperative muscle structures found during hardware removal procedures were similar to the muscle structures before the first operation. The radiographic outcome of fracture fixation was satisfactory.

**Conclusion:**

Surgery with PQ preservation is suitable for most distal radius fractures other than Types B1 and B2. For a small part of fractures involving the shaft of the radius, the PQ needed to be partially cut off to complete the operation. The postoperative muscle structures were close to normal.

## 1. Introduction

The distal radioulnar joint allows an individual to rotate the forearm to place the hand in a desired position to perform different tasks. The stability of the distal radioulnar joint is provided by the interaction between ligaments, muscles, and bones. The stabilizing structures are the triangular fibrocartilage complex, ulnocarpal ligament complex, extensor carpi ulnaris tendon and tendon sheath, pronator quadratus (PQ), interosseous membrane, and ligament, the bone itself, and the joint capsule [1]. Therefore, the main function of PQ muscles is forearm pronation. Since the distal radius fractures are the most common fractures of the upper extremity, with an incidence of 2 fractures per 1000 persons-years [2], studies on the PQ function confirmed that the superficial head participates in initial forearm pronation and the deep head provides the dynamic stability structure of the distal radioulnar joint [3]. The complex distal radius and unstable fractures are mainly treated through plate fixation [4]. To position the volar plate distal to the fracture site, the PQ muscle needs to be detached from its distal and radial side and lifted for optimal exposure to the fracture site [5]. With the standard volar approach, the PQ is typically elevated off the radial attachment. The distal part of the plate is often visible after PQ repair [6]. Because this surgery destroys the PQ, controversy about its repair persists [7,8]. The surgery in volar plate treatment with PQ preservation for distal radius fractures has been performed both locally and internationally [9,10]. The feasibility of this surgical method has been confirmed by anatomy research and clinical practice [11], but no reports on the clinical outcome of this method are available. This study discussed the surgical technique through trial surgery with PQ preservation on patients with distal radius fractures of different types.

## 2. Materials and methods

### 2.1. Subjects

This retrospective study included 46 patients with PQ preservation that received distal radius fracture surgery between August 2014 and October 2016. Arbeitsgemeinschaft für Osteosynthesefragen (AO) classification revealed that type A has four cases, type B3 has seven cases, type C1 has 10 cases, type C2 has 13 cases, and type C3 has 12 cases. Nineteen cases were male and 27 cases were female. The ages of the patients ranged from 29 to 62 years old, with an average age of 54 years old. All surgeries adopted dissection and locking plate fixation via the volar Henry approach [12]. The PQ muscles were preserved during surgery. If a PQ muscle affects reduction and fixation during surgery, the muscle would be partially cut off. The follow-up visits of these groups were for 12 months. This study was conducted in accordance with the Declaration of Helsinki and with approval from the Ethics Committee of Shandong University. Written informed consent was obtained from all participants.

### 2.2. Internal fixation surgery

**Figure 1:**
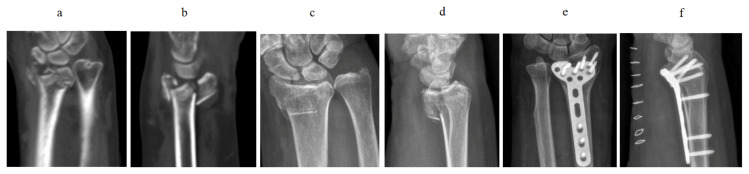
Patients whose pronator quadratus was preserved. a, b) preoperative CT scan showed a distal radius fracture of type C2 before the fixation operation. c, d) Preoperative X-Ray showed a distal radius fracture of type C1. e) One week after operation, the anterior and posterior films were displayed. f) One week after operation, lateral radiography was shown.

**Figure 2:**
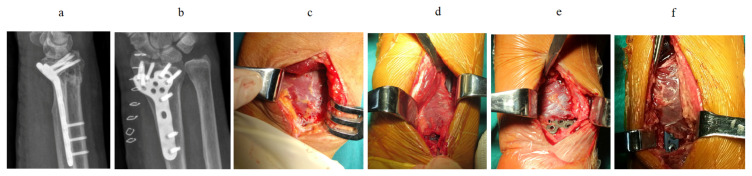
Patients whose pronator quadratus was preserved. a) one case of type C fracture was demonstrated by lateral radiography one week after operation. b) Anterior and posterior radiographs of 1 case of type B fracture one week after operation. c) Display of findings in 1 case of type C fracture after plate implantation. d) Display of findings in 1 case of type B fracture after plate implantation. e) Muscle condition of 1 case of type C fracture when the steel plate was removed in the second operation. f) Muscle condition of 1 case of type B fracture when the steel plate was removed in the second operation.

The internal fixation material was provided by Shandong Weigao Orthopaedic Materials Co., Ltd. (Jinan, China). All patients underwent preoperative computed tomography (CT) scan or X-ray to evaluate the fracture type (Figure 1a, Figure 1b, Figure 1c, Figure 1d). The radiation dose was 49.9 kv 5 mA/s; the location was wrist anterior-posterior position (Figure 1e, Figure 1f), and wrist lateral position (Figure 2a, Figure 2b). The Henry approach was applied in all participants, in which we isolated the wrist flexor and radial artery, pulled the radial flexor carpi radialis and the flexor pollicis longus toward the ulnar side, and pulled the radial artery toward the radial side to expose PQ. We identified the near and far edges of the muscle, slightly separated them outside the periosteum at the distal muscle edge, and pulled the muscles toward the proximal end to expose the fracture, which was then reduced under a C-arm X-ray and temporarily fixed with Kirschner wires (Weigao Orthopaedic Materials Co., Ltd, Jinan, China). The PQ was incised transversely at its distal portion and dissected off the periosteum using a periosteal elevator while preserving its ulnar and radial insertions to form the plate channel. The Locking Compression Plate was then inserted from the distal side to the proximal side to fully preserve the PQ. The C-arm X-ray was used to check the plate position, and screws were used to fix the plate (Figure 2c, Figure 2d). The muscle was dissected transversely and bluntly for approximately 5 mm. The screws were implanted by placing a guide. If the fracture cannot be exposed after pulling the muscle toward the proximal end, the PQ can be partially cut off from its end point of the radial site, followed by subperiosteal stripping and pulling toward the ulnar side to fully expose the fracture.

### 2.3. Postoperative management

On the first day postoperatively, the patients were encouraged to elevate their hands and begin early and unrestricted finger motion. The postoperative soft dressing was maintained for 14 days until the first follow-up visit. During this visit, the dressings and sutures were removed, radiographs were obtained, and the therapy was initiated by a certified hand therapist (Dr. X Zhong). Six weeks postoperatively, the patients were advanced to progressive strengthening and resistance exercises with the evidence of sufficient interval healing obtained from radiographs and clinical examination. All patient follow-ups proceeded with clinical examination and radiographs were obtained to confirm reduction and union at two, six, and 12 weeks postoperatively. The final visit was conducted 12 months postoperatively. All 14 patients (six males and eight females; mean age, 53 years; range, 29–61 years) who underwent internal fixation removal between June 2015 and January 2017 were selected to observe the structure of the PQ during hardware removal procedures.

## 3. Results

Positioning the plate and screw was easy in the surgical process of internal fixation via Henry approach with PQ preservation for distal radius fractures. The procedure would not increase the difficulty of the surgery if the operation is gentle and careful. However, it can completely preserve the integrity of PQ. In the four cases of type A, four cases of type B3, 10 cases of type C1, 12 cases of type C2, and 11 cases of type C3, PQ was preserved after completing the surgery. In the three cases of type B3 and one case of type C2, the fracture could not be entirely exposed after pulling the muscle to the near side, so we partially cut the muscle. One case of type C3 had severe muscle damage. Therefore, the PQ was not preserved.

From May 2014 to October 2016, a total of 232 cases of distal radius fractures were treated in our hospital. Of these, 175 cases underwent preoperative CT scans. The distance measured through preoperative CT scans between the fracture site and wrist joint was within 23.8 mm in 160 patients. Theoretically, surgery in which PQ was preserved was successfully conducted in 91% of the patients with distal radius fractures. In all of the four cases with partial PQ cut off on our trial basis, the distance between the fracture site and wrist joint exceeded 25 mm. The intraoperative finding showed that the proximal end of PQ can resist distal traction of up to 5 mm without causing muscle damage. In addition, the screws can be implanted in the distal side of the muscle by pulling the muscle itself. However, some patients may demonstrate severe muscle damage. Thus, the PQ cannot be preserved.

The radiographic outcome of fracture fixation is satisfactory (Figure 1e, Figure 1f, Figure 2a, Figure 2b). The radial inclination, radial height, and volar angulation were measured as described by Goldfarb et al. [13] and ulnar variance was measured as proposed by Medoff [14]. The average radial inclination, volar tilt, radial height, and ulnar variance from the neutral, and articular step-off were 19°, 5.6°, 10.6 mm, 0.2 mm, and articular 0.6 mm, respectively.

The wrist functions of the 41 patients were scored according to the Gartland–Werley scoring system [15]. A total of 31 cases showed excellent effects, six cases exhibited good effects, and four cases showed acceptable effects. The percentage of good cases was 90%. In the second surgery, we removed the internal fixation and found no significant muscle atrophy (Figure 2c, Figure 2d). The muscle structure found during hardware removal procedures was similar to the structure during the open reduction internal fixation procedures (Figure 2e, Figure 2f).

## 4. Discussion

We adopted the surgical technology that preserves PQ to treat 46 patients with distal radius fractures. The muscle in 41 patients was preserved after the surgery. Five patients underwent complete surgery in which PQ was cut off partially or completely. The mean operative time was 52 min. There were no instances of implant failure, adhesions requiring tenolysis, and tendon rupture. No patient developed carpal tunnel syndrome. All fractures healed without incision infection, radial artery injury, nerve damage, tendon rupture, and nonunion. All surgeries achieved satisfactory clinical results. Partial contusions were observed in the foreside of the PQ in one patient, but no cases wherein complete muscle rupture occurred were noted. According to the Gartland–Werley scoring system, mostly had excellent outcomes and no significant muscle atrophy was found after the removal of the internal fixation.

Muscle atrophy is a clinical problem after the surgical repair of a fracture and preserving the PQ was vital in distal radius fractures. Many studies showed that after distal radius fractures, there was consistent pronator strength loss or muscular dysfunction [16–19].

Recently, treatment of distal radius fractures through the volar approach has gained popularity. Several studies achieved good function scores and minimal complications [4,20,21]. The surgery of volar plate treatment with PQ preservation for distal radius fractures has been performed in many orthopaedic centres. However, no report on the clinical outcome of PQ preservation is available.

Recent anatomy studies have verified that the average mobility in the 13.1 mm range of the distal edge of the PQ of the lateral traction will not damage the muscle. Therefore, fractures of the distal radius in the 23.8 mm range can be exposed. Other studies have shown that when the plate is placed or adjusted under the PQ, it will not cause muscle fibre damage if the distance from the surface of the radius to the plate is less than 12 mm. If the plate length is greater than 52 mm, the use of the hole under the muscle [22] can be avoided. From May 2014 to October 2016, a total of 232 cases of distal radius fractures were treated in our hospital, wherein 175 cases underwent preoperative CT scan. The distance measured through preoperative CT scans between the fracture site and wrist joint was 23.8 mm among the 160 patients. Theoretically, surgery in which PQ was preserved was successfully conducted in 91% of the patients with distal radius fractures. In all of the four cases with partially cut-off PQ on our trial basis, the distance between the fracture site and wrist joint exceeded 25 mm. The intraoperative findings showed that the PQ on the proximal end can resist distal traction of up to 5 mm without causing any muscle damage. Therefore, the screws can be implanted in the distal side of the muscle by pulling the muscle. However, some patients demonstrated severe muscle damage, in which the PQ cannot be preserved.

Several studies have verified the feasibility of the technology. These studies presented the idea that preserving the PQ is practical and easy to implement [7,23,24]. Cannon et al. performed 28 operations and it proved the feasibility of the method and obtained similar short-term radiographic outcomes to the conventional volar plating approach [25].

Our current clinical observation revealed that the treatment with PQ preservation of the distal radius fractures by the Henry approach was suitable for all types of AO classification. Implantation of the plate and screw was easy. The PQ can be preserved if a careful operation is conducted. A few of the patients’ fracture sites extended to the shaft of the radius. In this case, the muscles needed to be partially cut off to complete the operation. The type B3 fracture site was often located in the proximal metaphysis of the stem and thus resulted in a high proportion of muscle cutting. This operation method cannot be applied to types B1 and B2 fractures, requiring other approaches.

Distal radius fracture surgery with PQ preservation possesses the following advantages. First, postoperative bleeding is reduced because the operation protects the lateral branch of the radial artery blood vessel without cutting the muscle. Second, postoperative functional exercise can be implemented early, which is beneficial to the recovery of the wrist joint function and reduces tendon adhesion [26]. Third, good preservation of the anatomical structure of PQ results in good preservation of muscle function. Fourth, the operative technique provides an anatomical basis for the application of PQ muscle-bone flap in the treatment of the necrosis of wrist disorder [27,28]. Fifth, preserving the radial attachment points of PQ can reduce damage to the blood supply around the fractures [29], leading to an increase in fracture healing rate. Sixth, the preservation of PQ anatomical structures can maintain the dynamic stability of the distal radioulnar joint [30], thereby reducing the incidence of wrist pain and discomfort after distal radial fractures. Cannon et al. presented a similar speculation [25].

Given these advantages, we believe that the operative technique can be clinically promoted. The exact measurement and rough assessment should be employed in the distance measurement between the fracture site and wrist joint through X-ray or preoperative CT. The fracture sites may be exposed by the distal traction in the operation process within a distance of 25 mm. If the distance exceeds 25 mm or severe damage to the muscle occurs, the operation may be implemented by partially or completely cutting off the PQ.

This study has limitations. The PQ cannot be preserved in fractures with severe muscle damage and the morphological difference of PQ may affect the operation. This operation also has a high demand for surgical skills and an operation by an inexperienced surgeon can cause iatrogenic injury. The decision to dissect PQ transversely or the traction for screw setting should be determined by the plate’s location, muscle damage, as well as the relationship between the muscle and screws. This technique requires further study by experienced surgeons and a large number of clinical research.
